# Supplementary material for: Using GIS Dashboards to highlight AMR data disparities in Africa for Policy, Research, and Public Health
Source: PLoS One. 2026 Sep 24;21(9):e0356734. doi: 10.1371/journal.pone.0356734 (PMC13606982; doi:10.1371/journal.pone.0356734)
Supplement: Supplementary file 1 [file pone.0356734.s001.pdf]

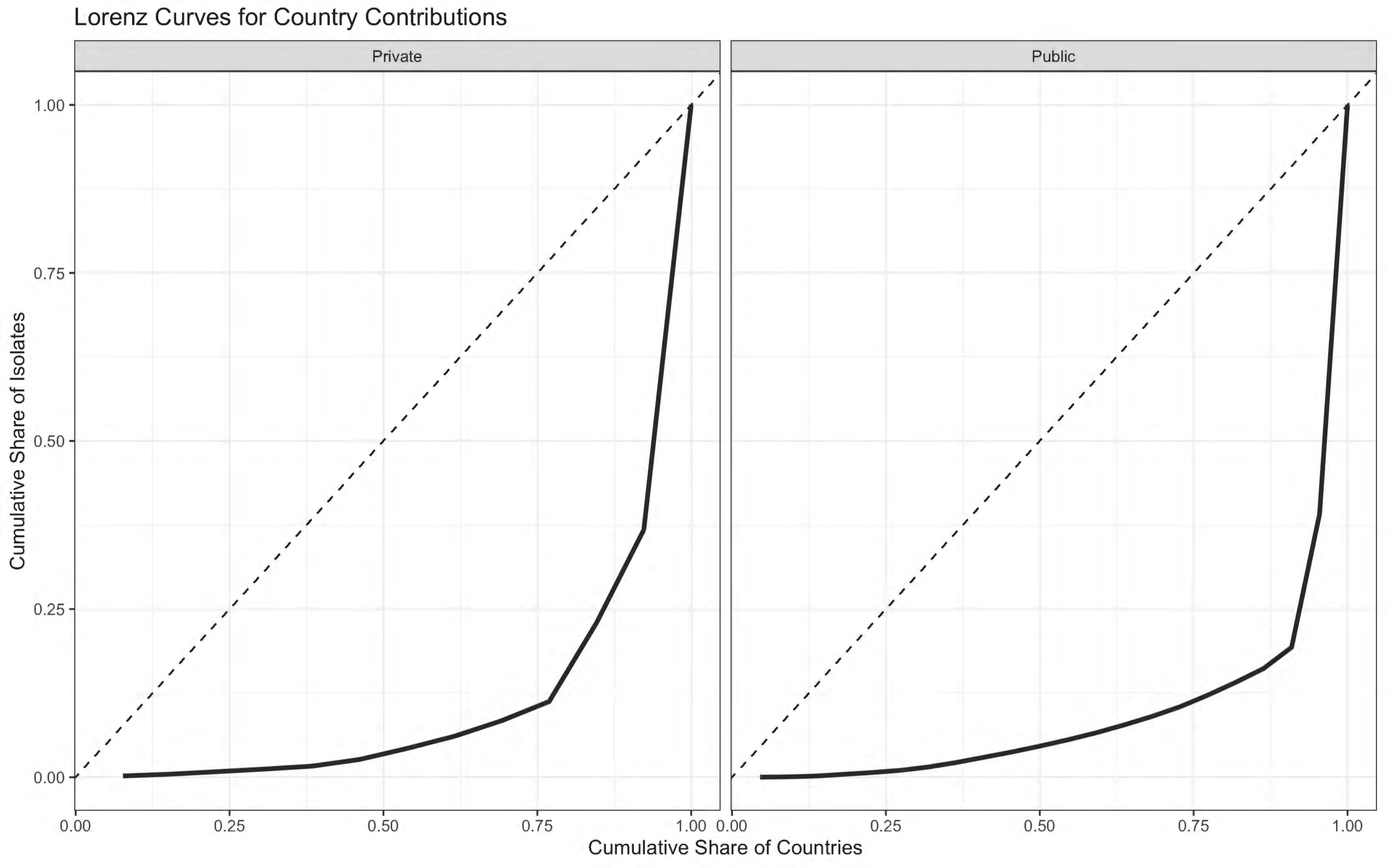

Lorenz Curves for Country Contributions
Private
Public
Cumulative Share of Isolates
Cumulative Share of Countries
0.00
0.25
0.50
0.75
1.00
