# Supplementary material for: Using GIS Dashboards to highlight AMR data disparities in Africa for Policy, Research, and Public Health
Source: PLoS One. 2026 Sep 24;21(9):e0356734. doi: 10.1371/journal.pone.0356734 (PMC13606982; doi:10.1371/journal.pone.0356734)
Supplement: Supplementary file 2 [file pone.0356734.s002.pdf]

A

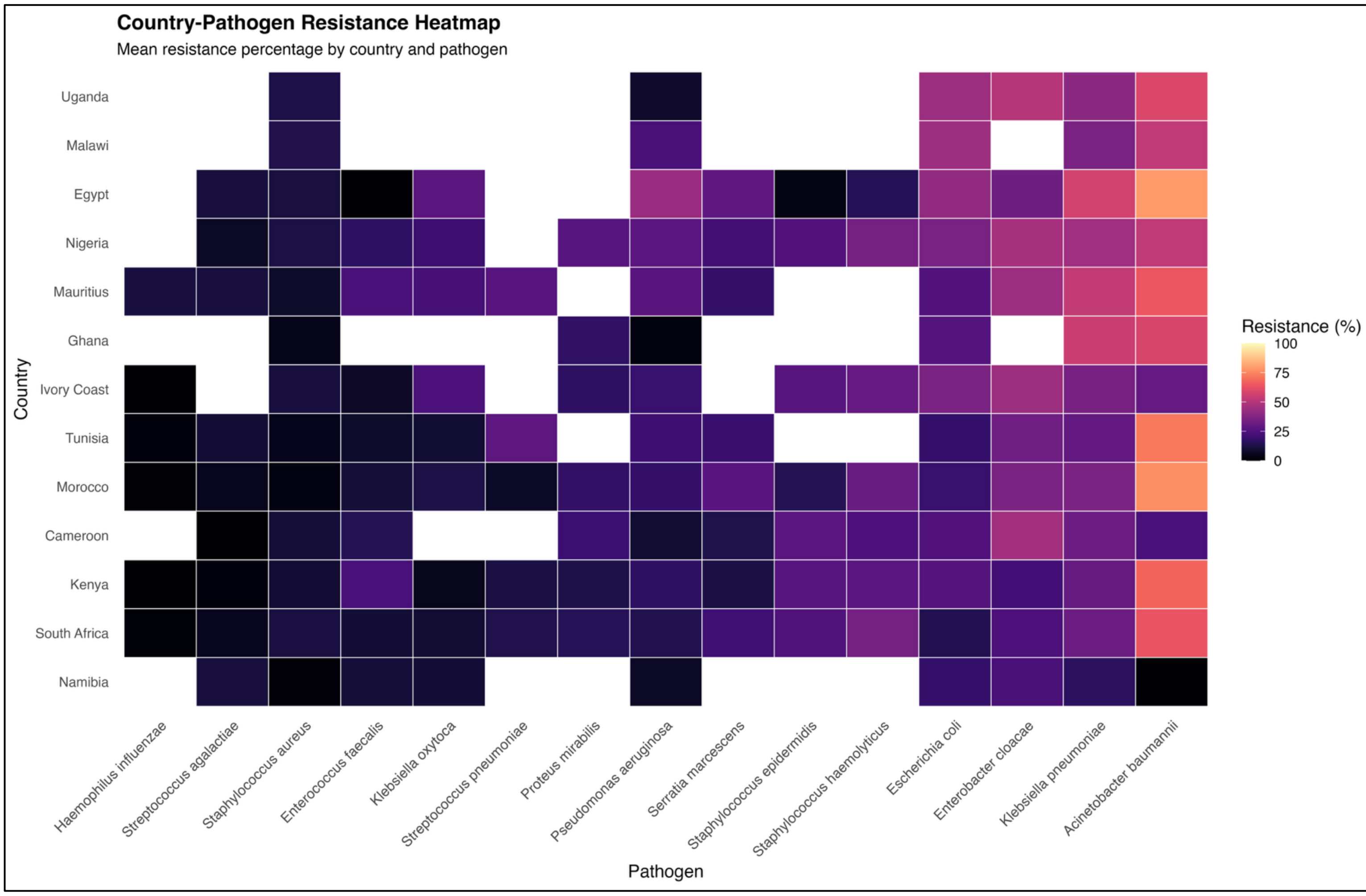
Country-Pathogen Resistance Heatmap
Mean resistance percentage by country and pathogen
Uganda
Malawi
Egypt
Nigeria
Mauritius
Ghana
Ivory Coast
Tunisia
Morocco
Cameroon
Kenya
South Africa
Namibia
Country
Haemophilus influenzae
Streptococcus agalactiae
Staphylococcus aureus
Enterococcus faecalis
Klebsiella oxytoca
Streptococcus pneumoniae
Proteus mirabilis
Pseudomonas aeruginosa
Serratia marcescens
Staphylococcus epidermidis
Staphylococcus haemolyticus
Escherichia coli
Enterobacter cloacae
Klebsiella pneumoniae
Acinetobacter baumannii
Pathogen
Resistance (%)
100
75
50
25
0

B

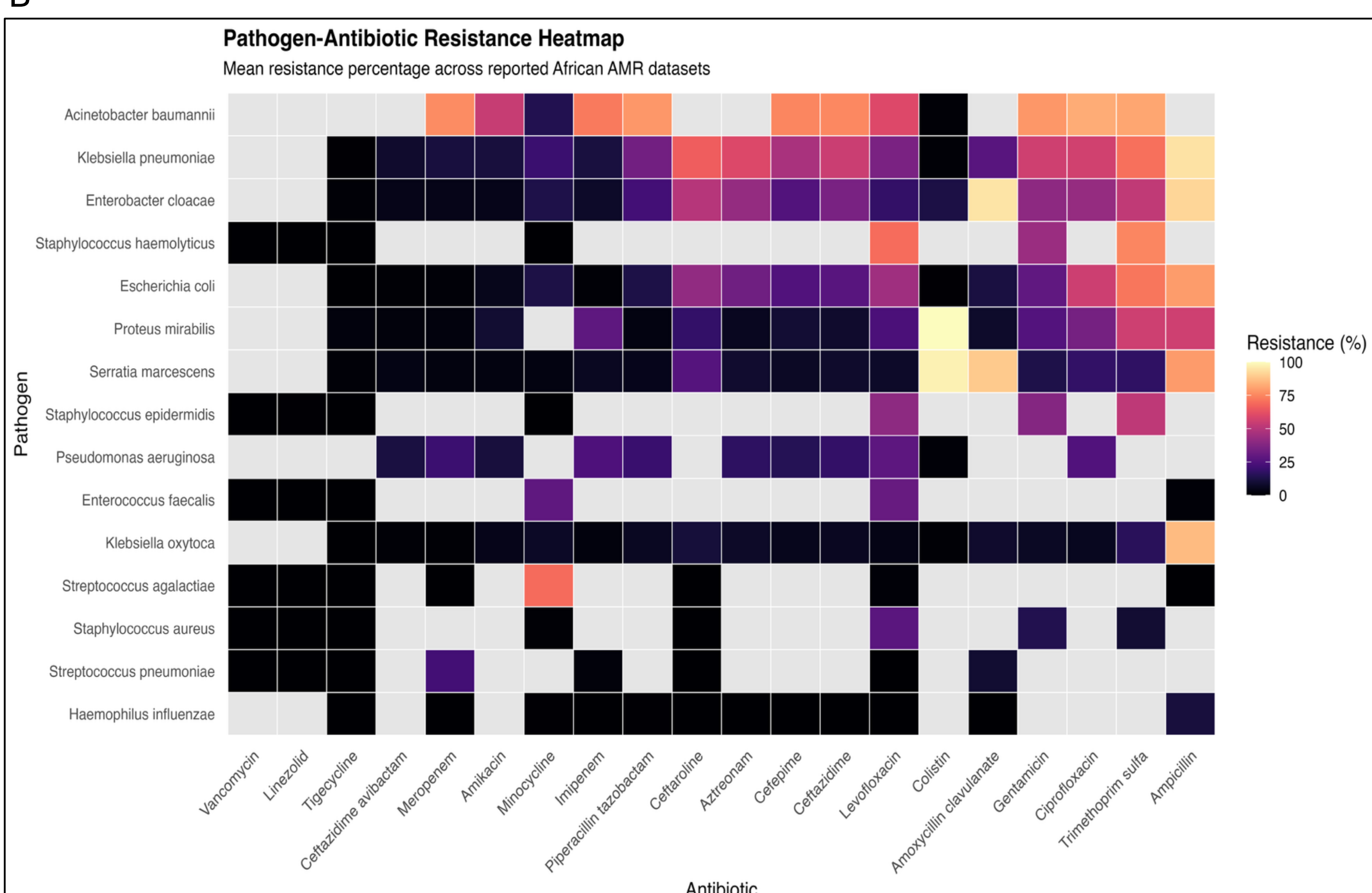
Pathogen-Antibiotic Resistance Heatmap
Mean resistance percentage across reported African AMR datasets
Acinetobacter baumannii
Klebsiella pneumoniae
Enterobacter cloacae
Staphylococcus haemolyticus
Escherichia coli
Proteus mirabilis
Serratia marcescens
Staphylococcus epidermidis
Pseudomonas aeruginosa
Enterococcus faecalis
Klebsiella oxytoca
Streptococcus agalactiae
Staphylococcus aureus
Streptococcus pneumoniae
Haemophilus influenzae
Pathogen
Vancomycin
Linezolid
Tigecycline
Ceftazidime avibactam
Meropenem
Amikacin
Minocycline
Imipenem
Piperacillin tazobactam
Ceftaroline
Aztreonam
Cefepime
Ceftazidime
Levofloxacin
Colistin
Amoxycillin clavulanate
Gentamicin
Ciprofloxacin
Trimethoprim sulfa
Ampicillin
Antibiotic
Resistance (%)
100
75
50
25
0
