# Supplementary material for: Using GIS Dashboards to highlight AMR data disparities in Africa for Policy, Research, and Public Health
Source: PLoS One. 2026 Sep 24;21(9):e0356734. doi: 10.1371/journal.pone.0356734 (PMC13606982; doi:10.1371/journal.pone.0356734)
Supplement: Supplementary file 3 [file pone.0356734.s003.pdf]

Supplementary Table S1. Unified Data Dictionary

This table explains every variable in the final harmonized dataset.

Example:

**Unified Variable Name**	**Description**	**Data Type**	**Example**	**Used in Figure/Analysis**
Country	Country where isolates were reported	Character	Uganda	Figure 1
Year	Year isolates were collected/reported	Numeric	2021	Figure 1
Isolate_Count	Number of isolates reported	Numeric	1543	Figure 1
Classification	Source category (Public or Private)	Character	Private	Figure 1
Source	Original contributing database	Character	ATLAS	Figure 1
Latitude	Country centroid latitude used for mapping	Numeric	1.3733	Figure 1
Longitude	Country centroid longitude used for mapping	Numeric	32.2903	Figure 1
Organism	Bacterial species name	Character	*E. coli*	Antibiotic analyses
Ciprofloxacin_I	Susceptibility interpretation for Ciprofloxacin	Character	Resistant	Resistance analysis
No Info	Indicates antibiotic not tested for isolate	Character	No Info	Antibiotic analyses
