# Supplementary material for: Using GIS Dashboards to highlight AMR data disparities in Africa for Policy, Research, and Public Health
Source: PLoS One. 2026 Sep 24;21(9):e0356734. doi: 10.1371/journal.pone.0356734 (PMC13606982; doi:10.1371/journal.pone.0356734)
Supplement: Supplementary file 4 [file pone.0356734.s004.pdf]

Supplementary Table S2. Variable Mapping Table

Table S2 shows how variables from different datasets were mapped into the harmonized schema.
Example:

Unified Variable	Public Dataset Variable	Private Dataset Variable	Transformation/Harmonization
Country	country_name	Country	Country names aligned manually
Year	collection_year	Year	Converted to numeric year
Isolate_Count	Isolates	isolate_total	Renamed into common variable
Classification	"Public" assigned	"Private" assigned	Manual classification
Source	MAAP	ATLAS	Retained original source
Organism	Organism	bacteria_name	Species naming harmonized
Ciprofloxacin_I	CIP_Result	Ciprofloxacin_I	Converted to S/I/R categories
Missing Antibiotic Values	NA	NULL	Replaced with "No Info"
