# Supplementary material for: Using GIS Dashboards to highlight AMR data disparities in Africa for Policy, Research, and Public Health
Source: PLoS One. 2026 Sep 24;21(9):e0356734. doi: 10.1371/journal.pone.0356734 (PMC13606982; doi:10.1371/journal.pone.0356734)
Supplement: Supplementary file 5 [file pone.0356734.s005.pdf]

Supplementary Table S3. Antibiotic Harmonization Table

This table explains how antibiotic names were aligned.

Example:

Unified Antibiotic Name	Public Dataset Name	Private Dataset Name
Ciprofloxacin	CIP	Ciprofloxacin
Ceftriaxone	CRO	Ceftriaxone
Gentamicin	GEN	Gentamicin
Trimethoprim sulfa	SXT	TMP/SMX
