# Supplementary material for: Using GIS Dashboards to highlight AMR data disparities in Africa for Policy, Research, and Public Health
Source: PLoS One. 2026 Sep 24;21(9):e0356734. doi: 10.1371/journal.pone.0356734 (PMC13606982; doi:10.1371/journal.pone.0356734)
Supplement: Supplementary file 6 [file pone.0356734.s006.pdf]

Supplemtary Table S4. Country-Level Distribution of Antimicrobial Resistance Surveillance Isolates

x	y	Country	Year	isolate	Classification	Source
12.614517	5.68008339	Cameroon	2020	199	Private	ATLAS
12.614517	5.68008339	Cameroon	2021	220	Private	ATLAS
12.614517	5.68008339	Cameroon	2022	158	Private	ATLAS
29.8361686	26.6060436	Egypt	2011	89	Private	ATLAS
29.8361686	26.6060436	Egypt	2012	121	Private	ATLAS
29.8361686	26.6060436	Egypt	2013	105	Private	ATLAS
-1.2369156	7.93932525	Ghana	2021	53	Private	ATLAS
-1.2369156	7.93932525	Ghana	2022	30	Private	ATLAS
-5.6120548	7.56143816	Ivory Coast	2020	135	Private	ATLAS
-5.6120548	7.56143816	Ivory Coast	2021	183	Private	ATLAS
-5.6120548	7.56143816	Ivory Coast	2022	203	Private	ATLAS
37.7913766	0.5971499	Kenya	2013	254	Private	ATLAS
37.7913766	0.5971499	Kenya	2014	318	Private	ATLAS
37.7913766	0.5971499	Kenya	2021	74	Private	ATLAS
37.7913766	0.5971499	Kenya	2022	46	Private	ATLAS
34.1971814	-13.194197	Malawi	2021	33	Private	ATLAS
34.1971814	-13.194197	Malawi	2022	26	Private	ATLAS
57.552152	_20.348404	Mauritius	2009	119	Private	ATLAS
-8.2864587	30.0885621	Morocco	2011	132	Private	ATLAS
-8.2864587	30.0885621	Morocco	2012	440	Private	ATLAS
-8.2864587	30.0885621	Morocco	2013	195	Private	ATLAS
-8.2864587	30.0885621	Morocco	2014	39	Private	ATLAS
-8.2864587	30.0885621	Morocco	2015	150	Private	ATLAS
-8.2864587	30.0885621	Morocco	2016	183	Private	ATLAS
-8.2864587	30.0885621	Morocco	2017	181	Private	ATLAS
-8.2864587	30.0885621	Morocco	2018	579	Private	ATLAS
-8.2864587	30.0885621	Morocco	2019	699	Private	ATLAS
-8.2864587	30.0885621	Morocco	2020	676	Private	ATLAS
-8.2864587	30.0885621	Morocco	2021	738	Private	ATLAS
-8.2864587	30.0885621	Morocco	2022	374	Private	ATLAS
17.1632021	-22.217052	Namibia	2008	87	Private	ATLAS
17.1632021	-22.217052	Namibia	2009	52	Private	ATLAS
7.99972176	9.5713441	Nigeria	2013	96	Private	ATLAS
7.99972176	9.5713441	Nigeria	2014	396	Private	ATLAS
7.99972176	9.5713441	Nigeria	2018	397	Private	ATLAS
7.99972176	9.5713441	Nigeria	2019	645	Private	ATLAS
7.99972176	9.5713441	Nigeria	2020	729	Private	ATLAS

7.99972176	9.5713441	Nigeria	2021	816	Private	ATLAS
7.99972176	9.5713441	Nigeria	2022	636	Private	ATLAS
24.9935818	-29.070148	South Africa	2004	3	Private	ATLAS
24.9935818	-29.070148	South Africa	2005	713	Private	ATLAS
24.9935818	-29.070148	South Africa	2006	725	Private	ATLAS
24.9935818	-29.070148	South Africa	2007	920	Private	ATLAS
24.9935818	-29.070148	South Africa	2008	102	Private	ATLAS
24.9935818	-29.070148	South Africa	2009	38	Private	ATLAS
24.9935818	-29.070148	South Africa	2011	306	Private	ATLAS
24.9935818	-29.070148	South Africa	2012	880	Private	ATLAS
24.9935818	-29.070148	South Africa	2013	912	Private	ATLAS
24.9935818	-29.070148	South Africa	2014	833	Private	ATLAS
24.9935818	-29.070148	South Africa	2015	797	Private	ATLAS
24.9935818	-29.070148	South Africa	2016	1216	Private	ATLAS
24.9935818	-29.070148	South Africa	2017	1310	Private	ATLAS
24.9935818	-29.070148	South Africa	2018	853	Private	ATLAS
24.9935818	-29.070148	South Africa	2019	1055	Private	ATLAS
24.9935818	-29.070148	South Africa	2020	1279	Private	ATLAS
24.9935818	-29.070148	South Africa	2021	1007	Private	ATLAS
24.9935818	-29.070148	South Africa	2022	704	Private	ATLAS
9.53287924	34.2227056	Tunisia	2010	1	Private	ATLAS
9.53287924	34.2227056	Tunisia	2011	265	Private	ATLAS
9.53287924	34.2227056	Tunisia	2012	291	Private	ATLAS
9.53287924	34.2227056	Tunisia	2013	193	Private	ATLAS
32.3577861	1.29666281	Uganda	2021	76	Private	ATLAS
32.3577861	1.29666281	Uganda	2022	47	Private	ATLAS
24.9935818	-29.070148	South Africa	2011	2	Private	DREAM
24.9935818	-29.070148	South Africa	2013	126	Private	DREAM
24.9935818	-29.070148	South Africa	2014	143	Private	DREAM
24.9935818	-29.070148	South Africa	2015	926	Private	DREAM
24.9935818	-29.070148	South Africa	2016	1245	Private	DREAM
24.9935818	-29.070148	South Africa	2017	1543	Private	DREAM
24.9935818	-29.070148	South Africa	2018	1190	Private	DREAM
24.9935818	-29.070148	South Africa	2019	753	Private	DREAM
24.9935818	-29.070148	South Africa	2018	185	Private	GEARS
24.9935818	-29.070148	South Africa	2019	175	Private	GEARS
24.9935818	-29.070148	South Africa	2020	145	Private	GEARS
29.8361686	26.6060436	Egypt	2017	444	Public	GLASS
29.8361686	26.6060436	Egypt	2018	2791	Public	GLASS

29.8361686	26.6060436	Egypt	2019	4508	Public	GLASS
29.8361686	26.6060436	Egypt	2020	1623	Public	GLASS
39.5461816	8.68479799	Ethiopia	2019	2121	Public	GLASS
46.6722265	-19.450886	Madagascar	2019	16573	Public	GLASS
34.1971814	-13.194197	Malawi	2019	1268	Public	GLASS
-3.5179813	17.3670571	Mali	2019	2048	Public	GLASS
35.4373877	-17.370132	Mozambique	2019	321	Public	GLASS
24.9935818	-29.070148	South Africa	2017	6142	Public	GLASS
24.9935818	-29.070148	South Africa	2018	19732	Public	GLASS
24.9935818	-29.070148	South Africa	2019	128583	Public	GLASS
24.9935818	-29.070148	South Africa	2020	334054	Public	GLASS
29.878866	16.0781364	Sudan	2017	32	Public	GLASS
29.878866	16.0781364	Sudan	2018	664	Public	GLASS
29.878866	16.0781364	Sudan	2019	283	Public	GLASS
9.53287924	34.2227056	Tunisia	2017	25052	Public	GLASS
9.53287924	34.2227056	Tunisia	2018	72194	Public	GLASS
9.53287924	34.2227056	Tunisia	2019	37735	Public	GLASS
9.53287924	34.2227056	Tunisia	2020	23979	Public	GLASS
32.3577861	1.29666281	Uganda	2017	1625	Public	GLASS
32.3577861	1.29666281	Uganda	2018	1400	Public	GLASS
32.3577861	1.29666281	Uganda	2019	4666	Public	GLASS
32.3577861	1.29666281	Uganda	2020	1535	Public	GLASS
27.7110758	-13.427408	Zambia	2019	1154	Public	GLASS
-1.7732919	12.3198835	Burkina Faso	2018	7106	Public	MAAP
-1.7732919	12.3198835	Burkina Faso	2019	441	Public	MAAP
12.614517	5.68008339	Cameroon	2017	3924	Public	MAAP
12.614517	5.68008339	Cameroon	2018	5771	Public	MAAP
12.614517	5.68008339	Cameroon	2019	5638	Public	MAAP
31.3952988	-26.491971	Eswatini	2016	1221	Public	MAAP
31.3952988	-26.491971	Eswatini	2017	1599	Public	MAAP
31.3952988	-26.491971	Eswatini	2018	1137	Public	MAAP
11.6876822	-0.6476207	Gabon	2016	1426	Public	MAAP
11.6876822	-0.6476207	Gabon	2017	2039	Public	MAAP
11.6876822	-0.6476207	Gabon	2018	1908	Public	MAAP
-1.2369156	7.93932525	Ghana	2016	2521	Public	MAAP
-1.2369156	7.93932525	Ghana	2017	2138	Public	MAAP
-1.2369156	7.93932525	Ghana	2018	2327	Public	MAAP
37.7913766	0.5971499	Kenya	2016	2088	Public	MAAP
37.7913766	0.5971499	Kenya	2017	2054	Public	MAAP

37.7913766	0.5971499	Kenya	2018	2130	Public	MAAP
34.1971814	-13.194197	Malawi	2016	1780	Public	MAAP
34.1971814	-13.194197	Malawi	2017	2562	Public	MAAP
34.1971814	-13.194197	Malawi	2018	2654	Public	MAAP
7.99972176	9.5713441	Nigeria	2016	3459	Public	MAAP
7.99972176	9.5713441	Nigeria	2017	6112	Public	MAAP
7.99972176	9.5713441	Nigeria	2018	4370	Public	MAAP
-14.511769	14.3622018	Senegal	2016	421	Public	MAAP
-14.511769	14.3622018	Senegal	2017	5772	Public	MAAP
-14.511769	14.3622018	Senegal	2018	5314	Public	MAAP
-11.795185	8.53271162	Sierra Leone	2016	32	Public	MAAP
-11.795185	8.53271162	Sierra Leone	2017	32	Public	MAAP
-11.795185	8.53271162	Sierra Leone	2018	44	Public	MAAP
34.7584758	-6.2783631	Tanzania	2016	834	Public	MAAP
34.7584758	-6.2783631	Tanzania	2017	2359	Public	MAAP
34.7584758	-6.2783631	Tanzania	2018	3165	Public	MAAP
32.3577861	1.29666281	Uganda	2016	5432	Public	MAAP
32.3577861	1.29666281	Uganda	2017	6494	Public	MAAP
32.3577861	1.29666281	Uganda	2018	4164	Public	MAAP
27.7110758	-13.427408	Zambia	2016	3199	Public	MAAP
27.7110758	-13.427408	Zambia	2017	3111	Public	MAAP
27.7110758	-13.427408	Zambia	2018	2696	Public	MAAP
29.788428	-18.929801	Zimbabwe	2016	563	Public	MAAP
29.788428	-18.929801	Zimbabwe	2017	1035	Public	MAAP
29.788428	-18.929801	Zimbabwe	2018	955	Public	MAAP
7.99972176	9.5713441	Nigeria	2014	1	Private	SIDERO-WT
24.9935818	-29.070148	South Africa	2013	2	Private	SIDERO-WT
24.9935818	-29.070148	South Africa	2014	3	Private	SIDERO-WT
24.9935818	-29.070148	South Africa	2015	3	Private	SIDERO-WT
24.9935818	-29.070148	South Africa	2016	9	Private	SIDERO-WT
9.53287924	34.2227056	Tunisia	2014	2	Private	SIDERO-WT
-1.2369156	7.93932525	Ghana	2017	2	Private	SOAR
37.7913766	0.5971499	Kenya	2016	14	Private	SOAR
37.7913766	0.5971499	Kenya	2017	21	Private	SOAR
37.7913766	0.5971499	Kenya	2018	9	Private	SOAR
-8.2864587	30.0885621	Morocco	2016	14	Private	SOAR
-8.2864587	30.0885621	Morocco	2017	9	Private	SOAR
7.99972176	9.5713441	Nigeria	2016	1	Private	SOAR
7.99972176	9.5713441	Nigeria	2017	3	Private	SOAR

9.53287924	34.2227056	Tunisia	2015	10	Private	SOAR
9.53287924	34.2227056	Tunisia	2016	77	Private	SOAR
9.53287924	34.2227056	Tunisia	2017	66	Private	SOAR

by Year, Data Source, Data Classification Across Africa
