# Supplementary material for: Using GIS Dashboards to highlight AMR data disparities in Africa for Policy, Research, and Public Health
Source: PLoS One. 2026 Sep 24;21(9):e0356734. doi: 10.1371/journal.pone.0356734 (PMC13606982; doi:10.1371/journal.pone.0356734)
Supplement: Supplementary file 7 [file pone.0356734.s007.pdf]

Supplementary Table S5. Published Antimicrobial Resistance Profiles Across Human, Animal, and One H

**Title**
Antibiotic Resistance of Salmonella in Poultry Farms of Mauritius
Blood culture surveillance in a secondary care hospital in Benin: epidemiology of bloodstream infection pa
Blood culture surveillance in a secondary care hospital in Benin: epidemiology of bloodstream infection pa
Blood culture surveillance in a secondary care hospital in Benin: epidemiology of bloodstream infection pa
Blood culture surveillance in a secondary care hospital in Benin: epidemiology of bloodstream infection pa
Blood culture surveillance in a secondary care hospital in Benin: epidemiology of bloodstream infection pa
Molecular detection of multidrug and methicillin resistance in Staphylococcus aureus isolated from wild p
Antimicrobial use and antimicrobial resistance in Escherichia coli in semi-intensive and free-range poultry
Antimicrobial use and antimicrobial resistance in Escherichia coli in semi-intensive and free-range poultry
Drug Resistance of Mycobacterium tuberculosis Complex in a Rural Setting, Angola
Drug Resistance of Mycobacterium tuberculosis Complex in a Rural Setting, Angola
Evaluation of antimicrobial resistance of Escherichia coli strains isolated from urinary tract infections in H
Methicillin-Resistant Staphylococcus aureus in the Community in Luanda, Angola: Blurred Boundaries w
Fluoroquinolone-Resistant Alcaligenes faecalis Related to Chronic Suppurative Otitis Media, Angola
Bacteria isolated from bloodstream infections at a tertiary hospital in Dar es Salaam, Tanzania – antimicro
Bacteria isolated from bloodstream infections at a tertiary hospital in Dar es Salaam, Tanzania – antimicro
Prevalence of antimicrobial resistance and its clinical implications in Ethiopia: a systematic review
Prevalence of antimicrobial resistance and its clinical implications in Ethiopia: a systematic review
Prevalence of antimicrobial resistance and its clinical implications in Ethiopia: a systematic review
Prevalence of antimicrobial resistance and its clinical implications in Ethiopia: a systematic review
Prevalence of antimicrobial resistance and its clinical implications in Ethiopia: a systematic review
Prevalence of antimicrobial resistance and its clinical implications in Ethiopia: a systematic review
Prevalence of antimicrobial resistance and its clinical implications in Ethiopia: a systematic review
Prevalence of antimicrobial resistance and its clinical implications in Ethiopia: a systematic review
Prevalence of antimicrobial resistance and its clinical implications in Ethiopia: a systematic review
Prevalence of antimicrobial resistance and its clinical implications in Ethiopia: a systematic review
Prevalence of antimicrobial resistance and its clinical implications in Ethiopia: a systematic review
Prevalence of antimicrobial resistance and its clinical implications in Ethiopia: a systematic review
Prevalence of antimicrobial resistance and its clinical implications in Ethiopia: a systematic review
Prevalence of antimicrobial resistance and its clinical implications in Ethiopia: a systematic review
Prevalence of antimicrobial resistance and its clinical implications in Ethiopia: a systematic review
Prevalence of antimicrobial resistance and its clinical implications in Ethiopia: a systematic review
Prevalence of antimicrobial resistance and its clinical implications in Ethiopia: a systematic review
Prevalence, Molecular Characterization, and Antimicrobial Susceptibility of Methicillin-Resistant Staphyl
Antibiotic resistance patterns in human, animal, food and environmental isolates in Ghana: a review
Antibiotic resistance patterns in human, animal, food and environmental isolates in Ghana: a review
Antibiotic resistance patterns in human, animal, food and environmental isolates in Ghana: a review
Antibiotic resistance patterns in human, animal, food and environmental isolates in Ghana: a review
Antibiotic resistance patterns in human, animal, food and environmental isolates in Ghana: a review

Antibiotic resistance patterns in human, animal, food and environmental isolates in Ghana: a review
Antibiotic resistance patterns in human, animal, food and environmental isolates in Ghana: a review
Antibiotic resistance patterns in human, animal, food and environmental isolates in Ghana: a review
Antibiotic resistance patterns in human, animal, food and environmental isolates in Ghana: a review
Antibiotic resistance patterns in human, animal, food and environmental isolates in Ghana: a review
Antimicrobial resistance pattern of avian pathogenic Escherichia coli with detection of extended-spectrum
Investigating the virulence genes and antibiotic susceptibility patterns of Vibrio cholerae O1 in environme
Investigating the virulence genes and antibiotic susceptibility patterns of Vibrio cholerae O1 in environme
Investigating the virulence genes and antibiotic susceptibility patterns of Vibrio cholerae O1 in environme
High rate of antibiotic resistance among pneumococci carried by healthy children in the eastern part of the Democratic Republic of the Congo
Serotype distribution and antimicrobial resistance of human Salmonella enterica in Bangui, Central Africa
Serotype distribution and antimicrobial resistance of human Salmonella enterica in Bangui, Central Africa
Serotype distribution and antimicrobial resistance of human Salmonella enterica in Bangui, Central Africa
Serotype distribution and antimicrobial resistance of human Salmonella enterica in Bangui, Central Africa
Serotype distribution and antimicrobial resistance of human Salmonella enterica in Bangui, Central Africa
Serotype distribution and antimicrobial resistance of human Salmonella enterica in Bangui, Central Africa
Serotype distribution and antimicrobial resistance of human Salmonella enterica in Bangui, Central Africa
Serotype distribution and antimicrobial resistance of human Salmonella enterica in Bangui, Central Africa
Serotype distribution and antimicrobial resistance of human Salmonella enterica in Bangui, Central Africa
Serotype distribution and antimicrobial resistance of human Salmonella enterica in Bangui, Central Africa
Antibiotics resistance in El Tor Vibrio cholerae 01 isolated during cholera outbreaks in Mozambique from
Antibiotics resistance in El Tor Vibrio cholerae 01 isolated during cholera outbreaks in Mozambique from
Antibiotics resistance in El Tor Vibrio cholerae 01 isolated during cholera outbreaks in Mozambique from
Antibiotics resistance in El Tor Vibrio cholerae 01 isolated during cholera outbreaks in Mozambique from
Methicillin-Resistant Staphylococcus aureus (MRSA): Prevalence and Antimicrobial Sensitivity Pattern

ealth Studies in Africa

Country	Author
Mauritius	Laxmee Phagoo and Hudaa Neetoo
Benin	Sien Ombelet et al.
Benin	Sien Ombelet et al.
Benin	Sien Ombelet et al.
Benin	Sien Ombelet et al.
Benin	Sien Ombelet et al.
South Africa	Trevor K. Wilson et al
Uganda	Irene Mbatidde et al
Uganda	Irene Mbatidde et al
Angola	Ariadna Rando-Segura et al
Angola	Ariadna Rando-Segura et al
Angola	Josefa Marlene Chindembele Camulombo et al
Angola	Teresa Conceição et al
Angola	Matuba Filipe et al
Tanzania	Moyo et al
Tanzania	Moyo et al
Ethiopia	Berhe et al
Ethiopia	Berhe et al
Ethiopia	Berhe et al
Ethiopia	Berhe et al
Ethiopia	Berhe et al
Ethiopia	Berhe et al
Ethiopia	Berhe et al
Ethiopia	Berhe et al
Ethiopia	Berhe et al
Ethiopia	Berhe et al
Ethiopia	Berhe et al
Ethiopia	Berhe et al
Ethiopia	Berhe et al
Ethiopia	Berhe et al
Ethiopia	Berhe et al
Ethiopia	Berhe et al
Ethiopia	Berhe et al
Egypt	M.A. Al-Ashmawy et al
Ghana	Pilar García-Vello et al
Ghana	Pilar García-Vello et al
Ghana	Pilar García-Vello et al
Ghana	Pilar García-Vello et al
Ghana	Pilar García-Vello et al

Ghana	Pilar García-Vello et al
Ghana	Pilar García-Vello et al
Ghana	Pilar García-Vello et al
Ghana	Pilar García-Vello et al
Ghana	Pilar García-Vello et al
Algeria	Chahrazed Aberkane et al.
Ghana	Abana et al.
Ghana	Abana et al.
Ghana	Abana et al.
Democratic Republic of Congo	Birindwa et al.
**Central African Republic**	Breurec S et al
**Central African Republic**	Breurec S et al
**Central African Republic**	Breurec S et al
**Central African Republic**	Breurec S et al
**Central African Republic**	Breurec S et al
**Central African Republic**	Breurec S et al
**Central African Republic**	Breurec S et al
**Central African Republic**	Breurec S et al
**Central African Republic**	Breurec S et al
**Central African Republic**	Breurec S et al
Mozambique	Dengo-Baloi et al.
Mozambique	Dengo-Baloi et al.
Mozambique	Dengo-Baloi et al.
Mozambique	Dengo-Baloi et al.
Eritrea	Eyob Yohaness Garoy et al.

**Journal**
J. World's Poult. Res. 5(3): 42-47
BMC Infect Dis 22, 119 (2022)
BMC Infect Dis 22, 119 (2022)
BMC Infect Dis 22, 119 (2022)
BMC Infect Dis 22, 119 (2022)
BMC Infect Dis 22, 119 (2022)
One Health 18 (2024) 100671
One Health 18 (2024) 100762
One Health 18 (2024) 100762
Emerging Infectious Diseases Vol. 24, No. 3, March 2018
Emerging Infectious Diseases Vol. 24, No. 3, March 2018
Rev. Cub. Cienc. Biol. 4 (2015) 71–77.
Microb. Drug Resist. 22 (2016) 22–27
Emerg. Infect. Dis. 23 (2017) 1740–1742
S. Afr. Med. J. 100 (2010) 835
S. Afr. Med. J. 100 (2010) 835
Resist. Infect. Control 10 (2021) 168
Resist. Infect. Control 10 (2021) 168
Resist. Infect. Control 10 (2021) 168
Resist. Infect. Control 10 (2021) 168
Resist. Infect. Control 10 (2021) 168
Resist. Infect. Control 10 (2021) 168
Resist. Infect. Control 10 (2021) 168
Resist. Infect. Control 10 (2021) 168
Resist. Infect. Control 10 (2021) 168
Resist. Infect. Control 10 (2021) 168
Resist. Infect. Control 10 (2021) 168
Resist. Infect. Control 10 (2021) 168
Resist. Infect. Control 10 (2021) 168
Resist. Infect. Control 10 (2021) 168
Resist. Infect. Control 10 (2021) 168
Resist. Infect. Control 10 (2021) 168
Resist. Infect. Control 10 (2021) 168
Foodborne Pathog. Dis. 13 (2016) 156–162
a review. PAMJ. 12 Feb 2020. 35(37)
a review. PAMJ. 12 Feb 2020. 35(37)
a review. PAMJ. 12 Feb 2020. 35(37)
a review. PAMJ. 12 Feb 2020. 35(37)
a review. PAMJ. 12 Feb 2020. 35(37)

a review. PAMJ. 12 Feb 2020. 35(37)
a review. PAMJ. 12 Feb 2020. 35(37)
a review. PAMJ. 12 Feb 2020. 35(37)
a review. PAMJ. 12 Feb 2020. 35(37)
a review. PAMJ. 12 Feb 2020. 35(37)
Veterinary World, 16(3): 449–454.
PLoS One. 2020; 15(7): e0236016.
PLoS One. 2020; 15(7): e0236016.
PLoS One. 2020; 15(7): e0236016.
BMC Pediatrics 2018 18:361
PLoS Negl Trop Dis 13(12): e0007917
PLoS Negl Trop Dis 13(12): e0007918
PLoS Negl Trop Dis 13(12): e0007919
PLoS Negl Trop Dis 13(12): e0007920
PLoS Negl Trop Dis 13(12): e0007921
PLoS Negl Trop Dis 13(12): e0007922
PLoS Negl Trop Dis 13(12): e0007923
PLoS Negl Trop Dis 13(12): e0007924
PLoS Negl Trop Dis 13(12): e0007925
PLoS Negl Trop Dis 13(12): e0007926
PLoS ONE 12(8): e0181496.
PLoS ONE 12(8): e0181496.
PLoS ONE 12(8): e0181496.
PLoS ONE 12(8): e0181496.
Canadian Journal of Infectious Diseases and Medical Microbiology Volume 2019, Article ID 8321834

Year	Species	Age	Source	One_Health
2015	Salmonella		Gut, Eggs, Intestine, litter	Animal
2022	Klebsiella pneumoniae	Less than 15	Blood	Human
2022	Salmonella Typhi	Less than 15	Blood	Human
2022	Staphylococcus aureus	Less than 15	Blood	Human
2022	Enterobacter cloacae	Less than 15	Blood	Human
2022	Escherichia coli	Less than 15	Blood	Human
2024	Staphylococcus aureus		Fecal	Animal
2024	Escherichia coli		Fecal - Free range	Animal
2024	Escherichia coli		Fecal - Semi intensive	Animal
2018	Mycobacterium tuberculosis		Sputum -Retreatment	Human
2018	Mycobacterium tuberculosis		Sputum - New cases	Human
2015	Escherichia coli		Urine	Human
2016	Staphylococcus aureus		Nasal	Human
2017	Alcaligenes faecalis		Nasal	Human
2010	Gram-positive		Blood	Human
2010	Gram-Negative		Blood	Human
2021	Staphylococcus aureus		Several	Human
2021	Streptococcus pneumoniae		Several	Human
2021	Streptococcus pyogenes		Several	Human
2021	Streptococcus agalactiae		Several	Human
2021	Coagulase-negative staphylococci		Several	Human
2021	Enterococcus spp.		Several	Human
2021	Klebsiella pneumoniae		Several	Human
2021	Klebsiella spp.		Several	Human
2021	Escherichia coli		Several	Human
2021	Enterobacter		Several	Human
2021	Proteus spp.		Several	Human
2021	Salmonella		Several	Human
2021	Shigella spp.		Several	Human
2021	Citrobacter		Several	Human
2021	Pseudomonas aeruginosa		Several	Human
2021	Acinetobacter		Several	Human
2021	Campylobacter		Several	Human
2016	Staphylococcus aureus		Milk	Animal
2020	Escherichia coli		Several	One-Health
2020	Staphylococcus spp		Several	One-Health
2020	Streptococcus spp		Several	One-Health
2020	Klebsiella spp		Several	One-Health
2020	Salmonella spp		Several	One-Health

2020	Mycobacterium spp		Several	One-Health
2020	Pseudomonas spp		Several	One-Health
2020	Neisseria spp		Several	One-Health
2020	Proteus spp		Several	One-Health
2020	Shigella spp		Several	One-Health
2023	Escherichia coli		Liver	Animal
2012	Vibrio cholerae		Not Known	Human
2014	Vibrio cholerae		Not Known	Human
2015	Vibrio cholerae		Not Known	Human
2018	Streptococcus pneumoniae		Nasal	Human
2019	*Salmonella enterica*			Human
2019	*Salmonella enterica*			Human
2019	*Salmonella enterica*			Human
2019	*Salmonella enterica*			Human
2019	*Salmonella enterica*			Human
2019	*Salmonella enterica*			Human
2019	*Salmonella enterica*			Human
2019	*Salmonella enterica*			Human
2019	*Salmonella enterica*			Human
2019	*Salmonella enterica*			Human
2017	Vibrio cholerae		Rectal swab	Human
2017	Vibrio cholerae		Rectal swab	Human
2017	Vibrio cholerae		Rectal swab	Human
2017	Vibrio cholerae		Rectal swab	Human
2019	Staphylococcus aureus		Pus	Human

Amikacin	Amoxycillin clavulanate	Ampicillin	Azithromycin	Cefepime	Cefoxitin
		40.00			
	81.00				
		30.20			
2.30					
0.00	41.70	73.50			
					33.30
	52.00	31.00			12.50
	25.00	62.00			10.00
		96.50			
		37.50			
		68.10			62.50
	26.00	70.00	47.00		
	65.00	38.00	28.00		8.00
	33.00	48.00		50.00	12.00
		9.00	15.00		
	23.00	57.00	48.00	23.00	29.00
	54.00	55.00	80.00		
13.00	61.00	93.00		53.00	43.00
10.00	57.00	75.00		23.00	8.00
10.00	52.00	83.00	32.00	35.00	37.00
7.00	64.00	66.00		48.00	50.00
8.00	64.00	77.00	44.00	38.00	50.00
	11.00	43.00			8.00
	20.00	84.00	34.00		27.00
49.00	70.00	74.00		37.00	55.00
11.00	78.00	86.00		56.00	42.00
66.00	44.00	60.00		85.00	
	36.00	53.00			
40.19		87.14			
0.00		86.90			
		66.80			
18.50		55.80			
0.00		93.50			

5.30		100.00			
		0.00			
5.45		81.00			
37.50		95.80			
		100.00			
0.00		0.00			
16.00		67.00			
11.00		72.00			
	4.00				
	1.00				
	3.00				
	0.00				
	3.00				
	2.00				
	0.00				
	0.00				
	2.00				
	0.00				
			100.00	0.00	
			100.00	0.00	
			100.00	0.00	
			100.00	39.00	

Ceftazidime	Ceftriaxone	Clarithromycin	Clindamycin	Erythromycin	Imipenem
				80.00	
	77.60				
	0.00				
				2.00	
	70.50				
	12.80				
			82.50	73.70	
				34.40	
				42.90	
41.00	32.00	70.00	25.00	45.00	
13.00	10.00				
10.00	26.00		25.00	22.00	
	28.00		24.00	20.00	
37.00	31.00	79.00	18.00	39.00	
100.00	19.00		27.00	37.00	
57.00	56.00		17.00	27.00	
63.00	40.00		31.00	76.00	2.00
52.00	38.00		23.00	71.00	5.00
70.00	40.00		56.00	80.00	
35.00	72.00		23.00	46.00	
	13.00			58.00	
	23.00			88.00	
19.00	32.00		57.00	73.00	
34.00	50.00		28.00	73.00	37.00
65.00	73.00		16.00	44.00	
	10.00		34.00	30.00	
13.30	26.90			100.00	
				7.30	
	4.19			12.67	
80.00	20.00			100.00	
0.00	0.00			100.00	

7.80	52.00				
	100.00			100.00	
29.80	41.40				
0.00	0.00	0.00		0.00	
3.00	2.00	21.00		69.00	
11.00	8.00	17.00		58.00	
			18.00	32.00	
				11.00	

Levofloxacin	Linezolid	Meropenem	Metronidazole	Minocycline	Penicillin
		0.00			
		0.00			
	0.00				77.60
		4.50			
		4.10			
	78.90				84.20
					96.00
82.60					
					68.90
					92.30
					82.00
					24.00
					17.00
					55.00
					43.00
		14.00			
		4.00			83.00
		4.00			78.00
		7.00			33.00
					46.00
					15.00
		39.00			49.00
		35.00			29.00
					87.90
					100.00
					90.20
					100.00
					100.00
					100.00

					100.00

Piperacillin tazobactam	Tigecycline	Vancomycin	Ampicillin sulbactam	Aztreonam
25.90				
		0.00		
16.30				
18.70				
		1.50		
		20.00		
	10.00			
	8.00			
52.00				97.00
43.00				96.00
44.00				97.00
				100.00
				100.00
33.00				59.00

			16.25	
	0.00			
	0.00			
	7.00			
		13.00		

Aztreonam avibactam	Cefixime	Ceftaroline	Ceftaroline avibactam	Ceftazidime avibactam
	26.00			

Ciprofloxacin	Colistin	Doxycycline	Daptomycin	Doripenem	Ertapenem	Gatifloxacin
39.60						
16.30		20.40				
72.70						
35.40						
15.80						
100.00						
42.50						
15.60						
26.00		38.00				
14.00		37.00				
13.00						
21.00		31.00				
33.00		64.00				
37.00		75.00				
25.00		67.00				
26.00		58.00				
13.00		35.00				
11.00		68.00				
9.00		59.00				
12.00		83.00				
25.00		70.00				
20.00		73.00				
61.00		67.00				
13.00		20.00				
19.40						
15.80						
2.80						
20.30						
0.00	0.00					

0.00						
11.40						
100.00	100.00					
35.40						
0.00						
87.50	14.37	91.87				
		0.00				
		27.00				
		14.00				
	0.00		100.00			
	0.00		11.00			
	0.00		0.00			
	0.00		0.00			

Gentamicin	Moxifloxacin	Oxacillin	Quinupristin dalfopristin	Sulbactam	Teicoplanin
0.00					
10.20		22.40			
68.20					
24.50					
1.00					
12.00					
26.00					
22.90					
29.00		47.00			
25.00		7.00			
50.00					
26.00		35.00			
26.00		69.00			
47.00	80.00				
42.00		98.00			
29.00	85.00	85.00			
26.00	78.00	50.00			
34.00	25.00	83.00			
11.00		100.00			
28.00					
33.00		31.00			
27.00	100.00	100.00			
61.00		100.00			
30.00					
26.10					
100.00					
77.80					
29.70					
16.90					

20.57					
25.60					
37.50					
32.50					
0.00					
2.00					
1.00					
		12.00			
1.20					

Tetracycline	Trimethoprim sulfa	Ceftolozane tazobactam	Cefoperazone sulbactam
100.00			
63.20			
52.00	28.00		
95.00	24.00		
	21.00		
64.80			
62.80			
53.00	44.00		
	40.00		
43.00	57.00		
71.00	11.00		
47.00	51.00		
61.00	26.00		
59.00	66.00		
56.00	62.00		
74.00	59.00		
51.00	48.00		
85.00	55.00		
34.00	30.00		
70.00	49.00		
68.00	65.00		
81.00	67.00		
56.00	67.00		
36.00	49.00		
65.20			
74.90	76.00		
41.90	0.00		
100.00	100.00		
82.60			
10.70	89.50		

85.40			
100.00			
82.50			
91.70	100.00		
100.00			
0.00			
13.00			
11.00			
25.00	6.00		
20.00			
22.00			
25.00			
28.00			
22.00			
13.00			
5.00			
6.00			
9.00			
7.00			
	25.00	100.00	
	32.00	100.00	
	44.00	0.00	
	100.00	100.00	

Meropenem vaborbactam	Cefpodoxime	Ceftibuten	Ceftibuten avibactam	Tebipenem
	100.00			

Temocilin	Amoxicillin	Chloramphenicol	cefuroxime	trimethoprim sulfamethoxazole
		60.00		
0.00	81.00	37.90	79.30	
	0.00			
7.00	0.00	65.90	72.70	16.30
11.00	41.70	24.50	40.80	
		100.00		
		44.30		
	69.00	40.00	26.00	
	23.00		11.00	
	24.00	29.00		
	50.00	12.00		
	57.00	42.00	48.00	
	33.00	30.00	46.00	
	83.00	53.00	50.00	
		45.00		
	71.00	36.00	44.00	
	64.00	35.00		
	68.00	48.00		
	63.00	20.00		
	85.00	45.00		
	77.00	61.00	50.00	
	88.00	53.00	33.00	
	86.00	39.00		
	80.00	18.00		
	55.60			
	100.00	49.10	50.30	
	75.00	45.90	60.90	
		100.00	100.00	
		73.90	33.30	
		100.00	32.00	

		84.60	78.80	
		0.00		
		90.40	51.40	
		100.00	16.70	
		27.50		62.50
		0.00	0.00	
		33.00	0.00	
		29.00	4.00	
	46.00	28.00		
	51.00	33.00		
	41.00	32.00		
	62.00	54.00		
	51.00	43.00		
	26.00	25.00		
	5.00	26.00		
	13.00	19.00		
	44.00	40.00		
	29.00	20.00		
			100.00	
			58.00	
			97.00	
			100.00	

Mezlocillin	Piperacillin	sulfamethoxazole-trimethoprim	Tobramycin	Rifampicin
				84.20
		52.60		
				87.00
			29.00	
			16.00	
			30.00	
			60.00	
			69.00	
			63.00	
			59.00	
			48.00	
			37.00	
			32.00	

Streptomycin	Eurofloxacin	Ceftiofur	Cephalexin	ESBL	Carbapenemase
80.00					
				79.30	0.00
				0.00	0.00
				68.20	4.50
				28.60	4.10
	6.30	6.00			
	56.00	4.00			
			59.60		
			66.00		
			38.00		
			67.00		
			12.00		
			53.00		
			100.00		
			78.00		
			33.00		
			89.00		

0.00					
69.00					
90.00					

**Carbapenems-fluoroqinoles**	**MDR**	**XDR**	**PDR**	**DCS**	**MRSA**	**oxacillin-ciprofloxacin**
0.00	87.90	32.80	0.00			
0.00	39.60	0.00	0.00	24.50		
	22.00				22.40	12.20
2.30	79.50	22.70	2.30			
2.00	69.40	8.20	2.00			
	71.10					
	8.00					
					23.30	

oxacillin-gentamicin	oxacillin-ciprofloxacin-gentamicin	oxacillin-doxycycline
10.20	10.20	8.20

**oxacillin-ciprofloxacin-doxycycline**	**oxacillin-trimethoprim-sulfamethox- azole**
6.10	2.00

oxacillin-erythromycin	Co-trimazole	Methicillin	Cephalothin	Cloxacillin	Nalidixic acid
2.00					
	25.00				
		35.00	41.00	78.00	45.00
		14.00		33.00	
					50.00
		28.00	15.00	66.00	33.00
		50.00			22.00
			76.00		
			36.00		
			49.00		
			65.00		
			69.00		
			18.00		
			63.00		
			55.00		
			59.00		
			7.00		
			89.00		
				75.90	
				100.00	
		63.80		4.70	
				0.00	
				100.00	

					95.00
					**93.00**
		72.00			

Nitrofurantoin	Norfloxacin	Cefotaxime	Cefpirome	Cephazolin	Aminopenicillin	Cefadroxil
22.00	26.00					
17.00	23.00					
	50.00	34.00				
20.00	19.00	34.00				
20.00	28.00	40.00				
48.00	42.00	56.00	50.00			
34.00	32.00	54.00	100.00	92.00		
14.00	21.00	45.00	100.00			
35.00	16.00	42.00		40.00		
36.00	17.00	50.00				
27.00	18.00	36.00				
	8.00					
57.00	26.00	42.00		100.00		
41.00	24.00	48.00				
29.00	9.00	68.00				
	11.00					
		8.76				100.00
		12.50				
		2.40				
		65.50				
		0.00			84.50	100.00

		45.50				
		0.00				
		38.20				
		12.50				
10.60						
25.00		0.00				

Cefotiam	Flucloxacilin	Ofloxacin	Cotrimoxazole	Ticarcillin	X	Y
					57.60	-20.20
					2.6	6.47
					2.6	6.47
					2.6	6.47
					2.6	6.47
					2.6	6.47
					28.19	-25.75
					32.58	0.35
					32.58	0.35
					13.23	-8.84
					13.23	-8.84
					13.23	-8.84
					13.23	-8.84
					13.23	-8.84
					35.00	-6.00
					35.00	-6.00
					38.75	9.02
					38.75	9.02
					38.75	9.02
					38.75	9.02
					38.75	9.02
					38.75	9.02
					38.75	9.02
					38.75	9.02
					38.75	9.02
					38.75	9.02
					38.75	9.02
					38.75	9.02
					38.75	9.02
					38.75	9.02
					38.75	9.02
					38.75	9.02
					31.77	30.03
100.00			81.20		-1.08	8.03
			54.50		-1.08	8.03
			100.00		-1.08	8.03
			82.40		-1.08	8.03
100.00			76.50		-1.08	8.03

					-1.08	8.03
			81.60		-1.08	8.03
			100.00		-1.08	8.03
			84.20		-1.08	8.03
			91.70		-1.08	8.03
		93.75			2.00	28.00
					**5.61**	**-0.21**
					**5.61**	**-0.21**
					**5.61**	**-0.21**
	73.00		75.00		15.31	-4.32
			41.00	46.00	20.94	6.61
			52.00	51.00	20.94	6.61
			44.00	41.00	20.94	6.61
			61.00	62.00	20.94	6.61
			51.00	51.00	20.94	6.61
			25.00	26.00	20.94	6.61
			3.00	5.00	20.94	6.61
			11.00	13.00	20.94	6.61
			44.00	44.00	20.94	6.61
			27.00	29.00	20.94	6.61
					34.75	-18.09
					34.75	-18.09
					34.75	-18.09
					34.75	-18.09
					39.45	15.61
